# Planktonic fungal community structures and their relationship to water quality in the Danjiangkou Reservoir, China

**DOI:** 10.1038/s41598-018-28903-y

**Published:** 2018-07-13

**Authors:** Zhaojin Chen, Jian Yuan, Feng Sun, Fei Zhang, Yan Chen, Chuanyu Ding, Jianwei Shi, Yuying Li, Lunguang Yao

**Affiliations:** 1 Collaborative Innovation Center of Water Security for Water Source Region of Mid-line of South-to-North Diversion Project of Henan Province, School of Agricultural Engineering, Nanyang Normal University, Nanyang, 473061 P.R. China; 2 School of Life Science and Technology, Nanyang Normal University, Nanyang, 473061 P.R. China; 3 Emergency Centre for Environmental Monitoring of the Canal Head of Middle Route Project of South-North Water Division, Xichuan, 474475 P.R. China

## Abstract

Planktonic fungi are important components of aquatic ecosystems, and analyses of their community composition and function have far-reaching significance for the ecological management and maintenance of reservoir environments. However, few studies have investigated the composition, distribution, and function of planktonic fungi in reservoir ecosystems and their relationship with water quality. Here, the composition of the planktonic fungal community in the surface water layer of the Danjiangkou Reservoir is investigated using Illumina MiSeq sequencing. According to the results, the reservoir community is primarily composed of 7 phyla, including Ascomycota, Rozellomycota, Basidiomycota, Chytridiomycota, and Zygomycota, comprising 294 genera, demonstrating the rich diversity of this community. Redundancy analysis (RDA) of the planktonic fungal community and environmental factors showed dissolved oxygen (DO), chemical oxygen demand (COD), total nitrogen (TN), chlorophyll a (Chl a), and permanganate (COD_Mn_) to be important factors influencing the distribution of planktonic fungi. Spearman correlation analysis of the planktonic fungal community composition and diversity indices with physical and chemical water quality parameters showed that the impacts of TN, COD and DO were the most significant. The results of this study on the planktonic fungal community in the Danjiangkou Reservoir area using high-throughput sequencing revealed that the community is sensitive to water quality parameters. This result provides a reference for studying the composition and distribution of the planktonic fungal community in Danjiangkou Reservoir and its role in the biogeochemical cycle.

## Introduction

The Middle Route of the South-to-North Water Diversion Project (MR-SNWDP) is a large-scale water diversion project to alleviate the serious shortage of water resources in northern China. Since the formal introduction of water on December 12, 2014, the water supply goals for three consecutive years have been met, with a total of 10.8 billion cubic meters of canal water diverted to northern China, which has resulted in excellent comprehensive economic, social, and ecological benefits^1^. The Danjiangkou Reservoir is the core water source area of the MR-SNWDP, and its water quality is directly related to drinking water safety for the residents of the areas receiving the water. Thus, it is of critical importance to carry out long-term monitoring of the water quality in this reservoir^2,3^.

Aquatic microorganisms are important components of these aquatic ecosystems, affecting biogeochemical processes in aquatic ecosystems, such as material circulation and pollutant release through catabolic and anabolic processes. Investigations of the community composition and function of aquatic microorganisms have far-reaching significance for the ecological management and maintenance of aquatic environments^4^. Aquatic microbes are sensitive to changes in water quality and are important indicators for biological monitoring and evaluating water quality^5–8^. Previous studies of aquatic microbes have primarily focused on the composition, distribution characteristics, and function of planktonic bacterial and archaeal communities and their relationship to water quality^9–13^. Conversely, relatively few studies have investigated planktonic fungi^14–16^. Nonetheless, the community structure of planktonic fungi has been shown to be affected by the water body type and its pH, temperature, and conductivity, as well as the physical and chemical properties of the organic matter present, such as nitrogen and phosphorus^5,17–19^. Cudowski *et al*. analyzed the planktonic fungal community of Augustów Canal using restriction fragment length polymorphism (RFLP) and observed that the physical and chemical properties of the body of water significantly affected the community composition, suggesting that planktonic fungi can be used to indicate water quality^5^. As most microorganisms cannot be cultured (>99%), modern culture-free molecular biological techniques have become an important means of studying microbial diversity. For example, Kagami *et al*. and Wang *et al*. used polymerase chain reaction denaturing gradient gel electrophoresis (PCR-DGGE) and cloning library methods, respectively, to study planktonic fungal communities^20,21^. However, compared with these methods, high-throughput sequencing can provide a more comprehensive picture of the composition of biomes due to its higher throughput for specific DNA fragments^22^. Thus, applying high-throughput sequencing for biological monitoring of water quality is receiving increasing attention^23^. Furthermore, this technique has been widely employed to evaluate the composition, distribution characteristics, and influencing factors of planktonic fungal communities^16,19^.

Our research group previously used high-throughput sequencing to examine the planktonic bacterial community in the Danjiangkou Reservoir area^24–26^, yet there are no reports to date on the planktonic fungal community in this water body. The present study focuses on the following: (1) monitoring of the water quality of the Danjiangkou Reservoir and providing data on water quality for MR-SNWDP; (2) clarifying the community composition and distribution characteristics of Danjiangkou Reservoir surface water planktonic fungi using high-throughput sequencing technology and contributing to follow-up study on biogeochemical cycle-related functions of these phytoplankton fungi; (3) investigating the relationship of Danjiangkou Reservoir planktonic fungal communities and physico-chemical parameters of water quality, which is expected to provide the basis for planktonic fungi as an indicator for changes in the water quality of reservoirs.

## Materials and Methods

### Study area and field sampling

**Figure 1:**
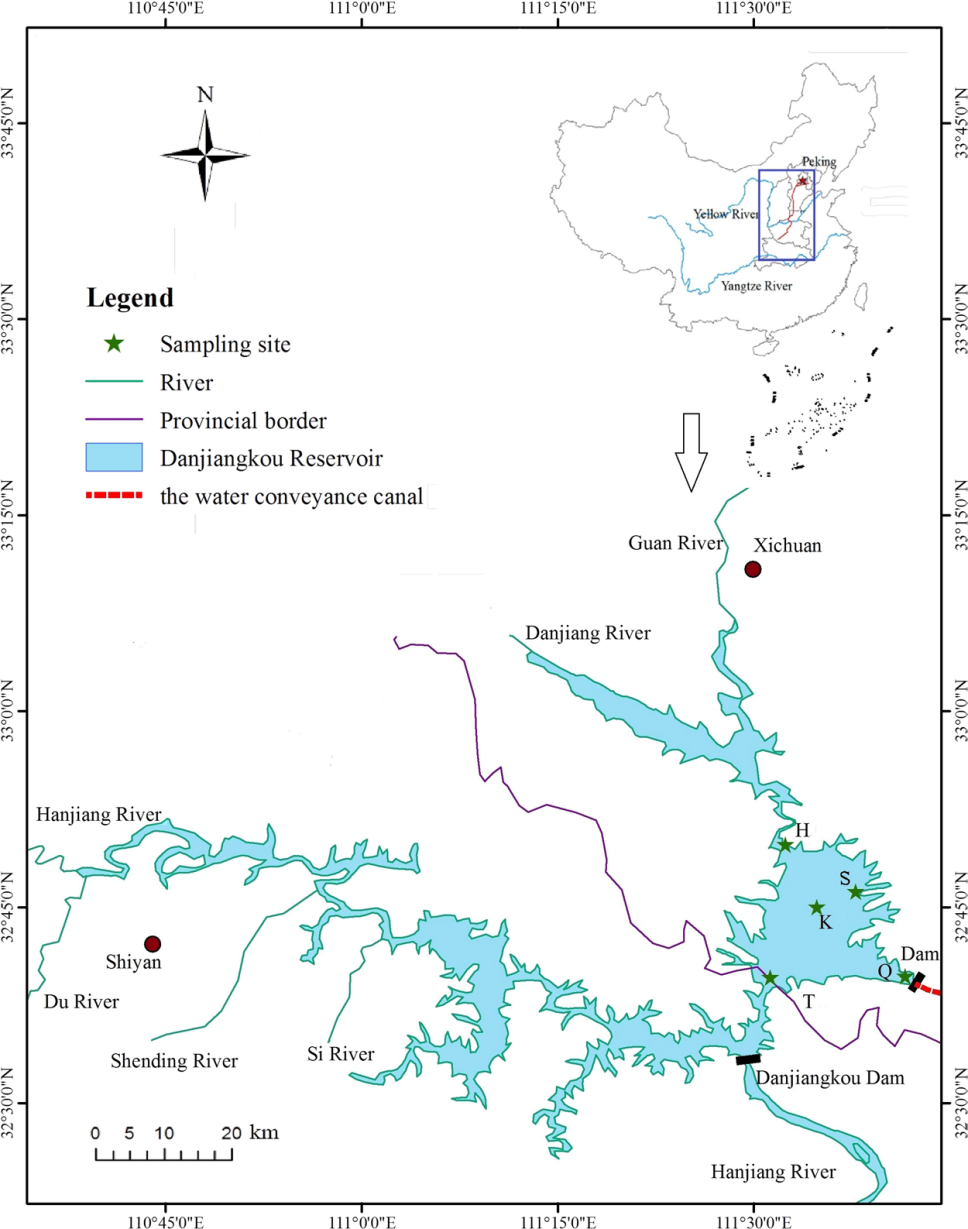
Locations of the five sampling stations in the Danjiangkou Reservoir and the water conveyance canal of Middle Route of the South-to-North Water Diversion Project (MR-SNWDP) in China. Station codes represent the first letter of the station’s name: K: Kuxin, Q: Qushou, S: Songgan, H: Heijizuo, T: Taizishan. The map was generated using ArcGIS 10.0 (ESRI, Redlands, CA, USA: http://www.esri.com/software/arcgis).

The Danjiangkou Reservoir (32°36′–33°48′N, 110°59′–111°49′E) is one of the largest river impoundments in the Yangtze River basin, located at the junction of the provinces of Henan and Hubei in central China (Fig. 1). It is the main water source of the MR-SNWDP, and responsible for the water supply of Beijing, Tianjin and more than 130 other cities in northern China. Therefore, the water quality in the Danjiangkou Reservoir is especially important for drinking water safety in these cities^27^. The first phase of the Danjiangkou Reservoir dam was completed in 1973, and the storage level was 157 m (water level above sea level); after the 2nd phase was completed in 2014, the storage level increased to 170 m.

Based on previous studies^24–26^, five typical ecological sites in the Danjiangkou Reservoir area were selected. At each station, water samples were collected in May 2017 at 0–50 cm below the water surface for high-throughput sequencing analysis and physico-chemical variables. Qushou (Q) station is 100 m upstream of the water outlet dam of the water conveyance canal of MR-SNWDP. Heijizuo (H) station is closer to the confluence of the Dan and Guan rivers, two major tributaries of the reservoir in Henan Province. Songgang (S) station is located in a reservoir bay that used to be influenced by shipping dock and tourism. Kuxin (K) station is located in the middle of the reservoir. Taizishan (T) station is in the confluence of the two sub-basins of the reservoir (Fig. 1). There were three replicates for each sample collected.

### Physico-chemical variables

Physico-chemical variables were measured according to the environmental quality standard for surface water of China (GB3838-2002). Water temperature (T), pH and dissolved oxygen (DO) were measured *in situ* using YSI 6920 (YSI Inc., Yellow Springs, Ohio, USA). Secchi depth (SD) was determined with a 30-cm-diameter Secchi disk. Water samples for chemical analysis were transported to the laboratory within 24 h, stored at 4 °C, and analyzed within one week of sample collection. The permanganate index (COD_Mn_) was calculated using the potassium permanganate index method, and chemical oxygen demand (COD) was measured using the potassium dichromate method. Total phosphorus (TP) was determined with acidified molybdate to form reduced phosphor-molybdenum blue, which was measured spectrophotometrically. Total nitrogen (TN) was assayed using alkaline persulfate digestion and UV spectrophotometry, ammonia nitrogen (NH_4_-N) was measured using a spectrophotometric method using Nessler’s reagent, and nitrate nitrogen (NO_3_-N) was measured using thymol spectrophotometry. The chlorophyll a (Chl a) concentration was estimated spectrophotometrically after extraction in 90% ethanol.

The trophic status of the Danjiangkou Reservoir area was assessed by measuring TN, TP, COD_Mn_, Chl a, and SD 5 per the improved Carlson’s trophic level index (TLI)^28^. The detailed evaluation method and classifications are shown in the Supplementary material and Table S1.

### DNA extraction and sequencing

For the MiSeq sequencing analysis of planktonic fungal communities, 800 mL of surface water was pre-filtered through a 200-μm sieve to remove debris and meso- and macroplankton and then filtered through a 0.22-μm pore polycarbonate membrane (Millipore, Billerica, MA, USA)^29^. Planktonic fungal genomic DNA captured by the stored filters was extracted using the E.Z.N.A.^®^. Water DNA Kit (Omega Bio-Tek, Norcross, GA, USA) following the manufacturer’s instructions. The internal transcribed spacer (ITS) region of the fungal rRNA gene was amplified using ITS1F (CTTGGTCATTTAGAGGAAGTAA) and 2043 R (GCTGCGTTCTTCATCGATGC). The PCR conditions used were as follows: 95 °C for 3 min; followed by 45 cycles of 95 °C for 10 s (denaturation), 55 °C for 30 s (annealing), 72 °C for 30 s (elongation)^30^. The PCR assays were performed in a 20 μL mixture containing 4 μL of 5× FastPfu buffer, 2 μL of 2.5 mM deoxyribonucleotide triphosphates (dNTPs), 0.8 μL of each primer (5 μM), 0.4 μL of FastPfu Polymerase, 10 ng of template DNA, and Milli-Q water. PCR was performed in triplicate for each sample, and the products were purified using AxyPrepDNA Gel Extraction Kit (Axygen Biosciences, Union City, CA, US) and re-quantified with QuantiFluor™ ST (Promega, Madison, WI, USA). Sequencing was performed by Shanghai Majorbio Bio-Pharm Technology Co., Ltd. (Shanghai, China) using the Illumina MiSeq PE300 platform^31^.

### Pyrosequencing data

Sequence data were processed using Quantitative Insights Into Microbial Ecology (QIIME, version 1.7.0). Operational taxonomic units (OTUs) were clustered with a 97% similarity cutoff using UPARSE (version 7.1). The taxonomy of each ITS gene sequence was analyzed using the RDP Classifier (http://rdp.cme.msu.edu/) against the UNITE fungal database at 75% similarity^32^. The OTU number of each sample was used to represent species richness. Rarefaction curves and Shannon-Wiener indices were generated, and ACE, Shannon, and Chao1 estimators were calculated to compare fungal richness and diversity. We used the unweighted UniFrac distance for principal coordinate analysis (PCoA) and the unweighted pair group method with arithmetic mean (UPGMA) clustering.

### Statistical analyses

Detrended correspondence analysis (DCA) for biological data was applied to determine whether to use linear or unimodal ordination methods. DCA of the MiSeq sequencing OTU data revealed that longest gradient length was less than 3.0; therefore, we chose redundancy analysis (RDA) using Canoco 4.5 to examine the relationship between planktonic fungal community and environmental factors. Spearman correlation analysis was performed between the fungal community composition and diversity indices and the physico-chemical water quality parameters.

The treatment means were compared by one-way analysis of variance (ANOVA) (*P* < 0.05) in SPSS v. 19.0 for Windows.

## Results

### Evaluation of the water quality and trophic status of the Danjiangkou Reservoir

Long-term monitoring results revealed that the water quality of Danjiangkou Reservoir was generally good. However, agricultural nonpoint source pollution, industrial wastewater, and domestic sewage from villages and towns were present, resulting in TN values that notably exceeded the standard limit^27,33^. The water quality monitoring results for Danjiangkou Reservoir in May 2017 are consistent with the above findings, as the water quality at the five monitoring sites was generally good. All indicators, with the exception of TN, met the Grade II water quality standards for the Environmental Quality Standards for Surface Water (GB38382-2002) (Table 1). The TN content at Taizishan and Songgang exceeded 1.00 mg/L, which is the standard limit for Grade IV surface water, and the TN content at Qushou, Kuxin, and Heijizui ranged from 0.50–1.00 mg/L, which is the standard limit for Grade II surface water. The COD_Mn_, COD, Chl a, TP, TN, and NO_3_-N levels at Taizishan were higher than those at other monitoring sites, and the differences in COD_Mn_, TN, NO_3_-N and Chl a were significant (*P* < 0.05) or extremely significant (*P* < 0.01) (Table 1).

**Table 1:** Main physicochemical characteristics and trophic level index (TLI) of water samples (means ± S.E.).

Station	T (°C)	pH	DO (mg/L)	SD (m)	COD_Mn_ (mg/L)	COD (mg/L)	TP (mg/L)	TN (mg/L)	NH_4_-N (mg/L)	NO_3_-N (mg/L)	Chl a (mg/m^3^)	TLI	Trophic state	Water quality
K	16.60 ± 0.36	8.47 ± 0.14b	8.69 ± 0.07ab	5.000 ± 0.020e	3.200 ± 0.100a	14.30 ± 0.27d	0.013 ± 0.006ab	0.947 ± 0.032c	0.030 ± 0.003a	0.76 ± 0.05c	0.50 ± 0.02ab	28.16	Oligotrophic	Excellence
Q	16.87 ± 0.65	8.47 ± 0.08b	9.77 ± 0.14c	2.997 ± 0.006b	3.767 ± 0.058c	11.37 ± 0.40ab	0.023 ± 0.006b	0.907 ± 0.012b	0.053 ± 0.003b	0.70 ± 0.02b	0.60 ± 0.02b	33.00	Mesotrophic	Good
S	16.68 ± 0.55	8.64 ± 0.09c	8.49 ± 0.06a	2.900 ± 0.007a	3.433 ± 0.058b	12.13 ± 0.60bc	0.023 ± 0.006b	1.013 ± 0.006d	0.146 ± 0.005d	0.51 ± 0.03a	0.42 ± 0.02a	31.97	Mesotrophic	Good
H	16.71 ± 0.90	8.21 ± 0.04a	9.57 ± 0.25c	4.297 ± 0.006d	3.200 ± 0.025b	11.27 ± 0.42a	0.010 ± 0.04a	0.780 ± 0.030a	0.021 ± 0.001a	0.66 ± 0.02b	0.56 ± 0.02b	28.52	Oligotrophic	Excellence
T	17.35 ± 0.40	8.46 ± 0.02b	8.79 ± 0.17b	4.203 ± 0.006c	4.433 ± 0.058e	12.73 ± 0.40c	0.023 ± 0.006b	1.213 ± 0.006e	0.069 ± 0.009c	1.07 ± 0.01d	1.21 ± 0.14c	35.41	Mesotrophic	Good

Means within the same column followed by the same letter are not significantly different at *P* < 0.05, as based on one-way ANOVA.

Determining the trophic status is a key part of ecologically monitoring and evaluating the Danjiangkou Reservoir^34^. The TLI index of the Danjiangkou Reservoir area ranged from 28.16 to 35.41, with an oligotrophic status observed for Kuxin and Heijizui and a mesotrophic status observed for Qushou, Songgang, and Taizishan (Tables 1 and S1). The overall water quality of the Danjiangkou Reservoir was good (Table 1).

### Sequencing results and diversity indices

**Figure 2:**
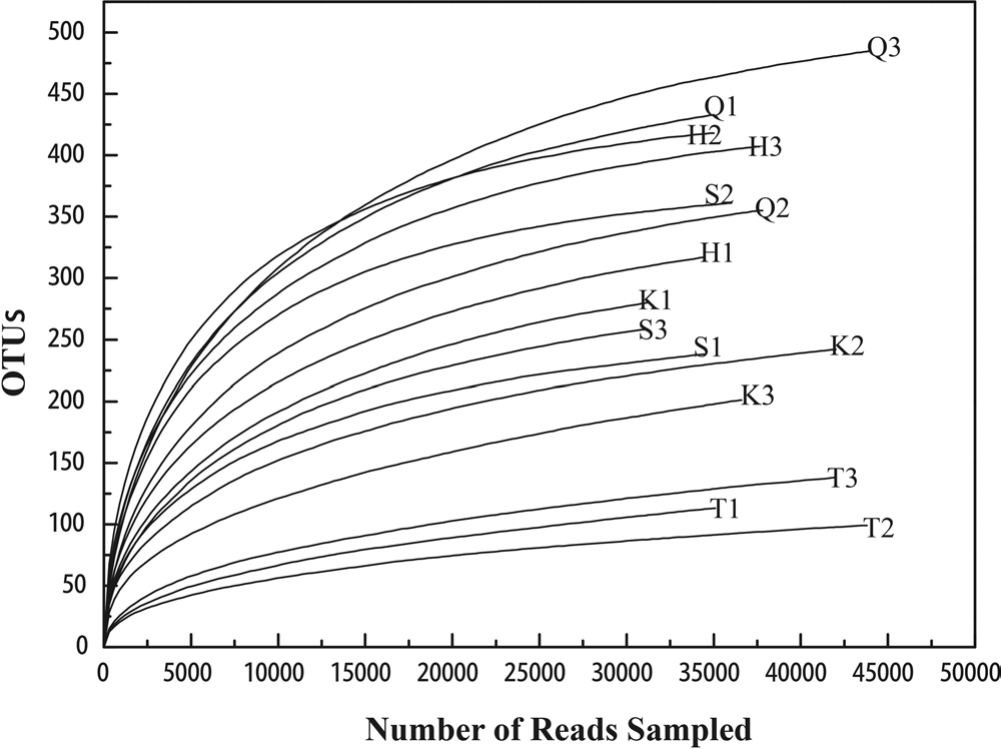
Rarefaction curves base on pyrosequencing of planktonic fungal communities.

Our high-throughput sequencing results showed an average number of sequence reads of 37,077 and an average OTU number of 290 for the samples from the five monitoring sites (Table 2). A rarefaction curve of planktonic fungi in the Danjiangkou Reservoir area is shown in Fig. 2. Species diversity increased as the sequencing coverage increased, and the number of species tended to be constant when the number of sequencing reads was more than 15,000. In addition, Good’s coverage was over 99.80% for the libraries prepared from all five monitoring site samples, indicating high sequence coverage for these libraries (Table 2).

**Table 2:** MiSeq sequencing results and diversity estimates for each sampling site (means ± S.E.).

Station	Reads	OTUs	Shannon	Simpson	Ace	Chao1	Coverage (%)
K	36600 ± 5400	241 ± 40b	1.74 ± 0.47ab	0.48 ± 0.14b	338.34 ± 37.60ab	301.27 ± 15.46b	99.81 ± 0.040a
Q	38948 ± 4613	424 ± 65d	2.86 ± 0.26 cd	0.15 ± 0.04a	477.61 ± 61.33c	467.02 ± 61.10c	99.80 ± 0.020a
S	33877 ± 2472	286 ± 66bc	2.35 ± 0.43bc	0.30 ± 0.09a	321.03 ± 54.87ab	311.89 ± 57.51b	99.85 ± 0.040ab
H	35713 ± 1675	381 ± 55 cd	3.12 ± 0.49d	0.15 ± 0.07a	419.23 ± 37.99bc	407.90 ± 44.53c	99.83 ± 0.030ab
T	40245 ± 4624	117 ± 20a	1.54 ± 0.04a	0.29 ± 0.01a	247.24 ± 66.04a	185.15 ± 40.27a	99.88 ± 0.030b

Means within the same column followed by the same letter are not significantly different at *P* < 0.05, as based on one-way ANOVA.

The planktonic fungal community in the Danjiangkou Reservoir was also examined using Chao1 and ACE community abundance indices, Shannon and Simpson community diversity indices, and library coverage. Based on the results, the samples from the five monitoring sites exhibited high fungal abundance and community diversity (Table 2). Based on OTU number, Chao1 and ACE abundance indices, Shannon and Simpson diversity indices, and library coverage, the community diversity of the planktonic fungi in the samples from the five monitoring sites was as follows: Qushou > Heijizuo > Songgang > Kuxin > Taizishan. Among the samples, OTU number, Chao1 and ACE abundance indices, and the Shannon index for Taizishan were lower than those of the other samples; the differences in OTU number and Chao1 were significant (*P* < 0.05), indicating that the diversity of the planktonic fungal community at Taizishan was significantly lower than that at the other sites (Table 2).

### Patterns of fungal beta diversity in the Dangjiangkou Reservoir

**Figure 3:**
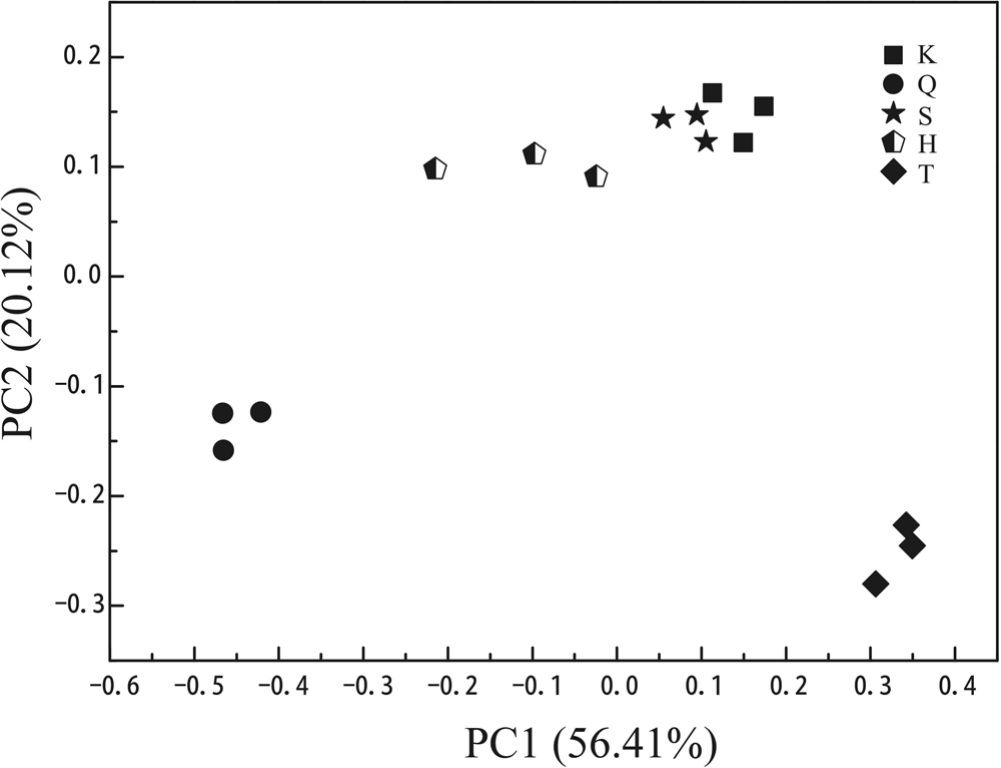
PCoA plot of the samples using the unweighted UniFrac distance metric.

Both principal coordinate analysis (PCoA) and unweighted pair group method with arithmetic mean (UPGMA) clustering analyses can describe community differences among samples, whereby a close distance between two samples indicates a similar species composition. The results of PCoA analysis (OTU level) of planktonic fungi for the samples from the five monitoring sites in the Danjiangkou Reservoir area are shown in Fig. 3. The variances explained by axes one and two were 56.41% and 20.12%, respectively. The community results for the samples from Kuxin and Songgang were most similar, clustering in the upper right of the graph, and the sample from Heijizui was distributed in the upper middle of the graph. The communities in samples from Qushou and Taizishan differed greatly from those of the other sites and were distributed in the lower left and lower right lower of the graph, respectively (Fig. 3). The UPGMA method based on unweighted UniFrac was used for clustering analysis of similarities in the composition of the planktonic fungal communities, and the results were similar to those of PCoA analysis (Fig. S1). At a similarity level of 0.209, the planktonic fungal composition of the samples from the five monitoring sites could be divided into four groups, with the samples from Kuxin and Songgang being similar and clustering into the same group. The samples from Heijizui, Taizishan, and Qushou self-clustered within corresponding individual groups. In contrast, the samples from Taizishan and Qushou were far from other samples in the dendrogram, indicating that their fungal communities differed greatly from those of the other samples.

### Analysis of the community structure of planktonic fungi

**Figure 4:**
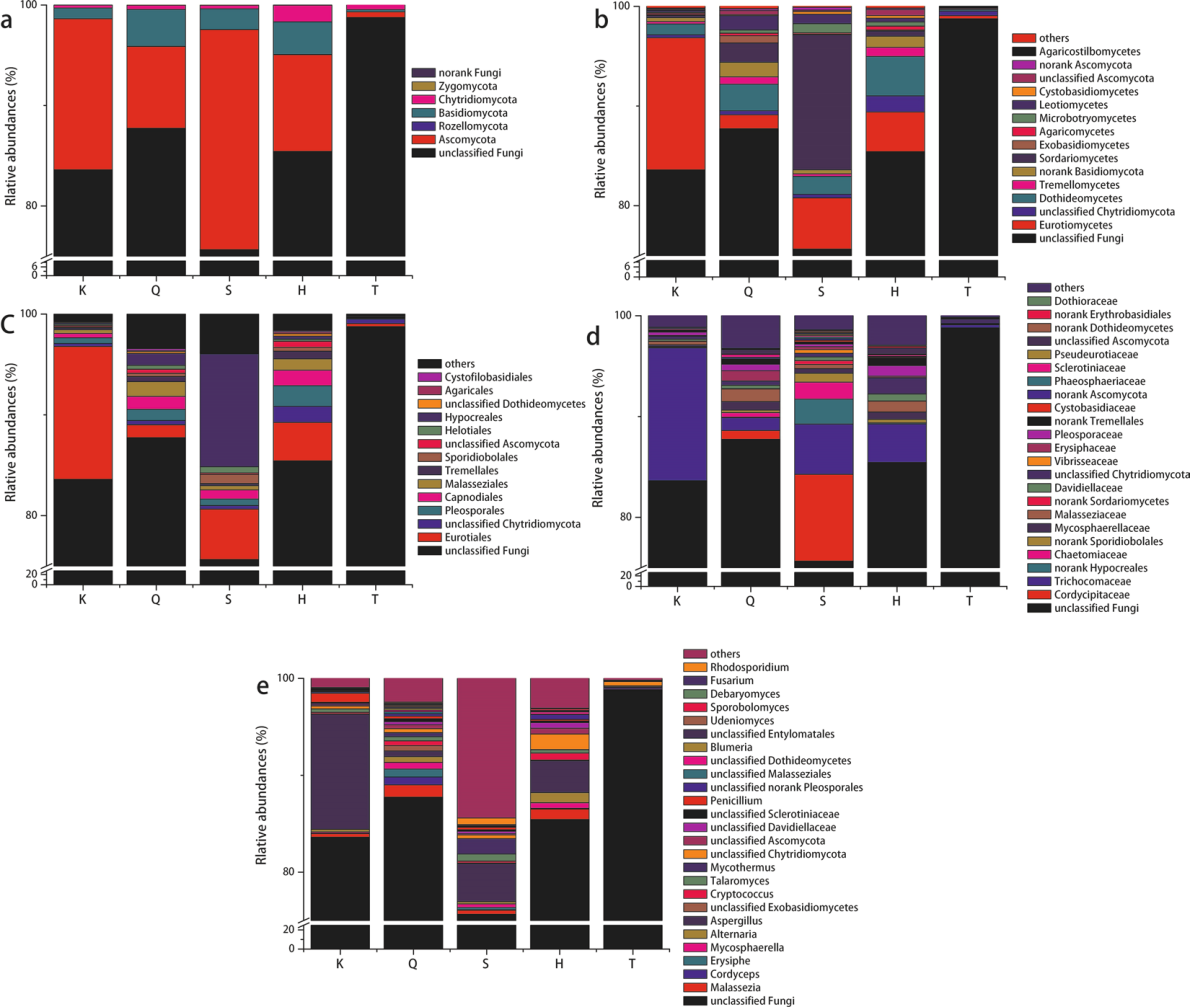
Relative abundance of planktonic fungal sequences at the phylum (**a**) class (**b**) order (**c**) family (**d**) and genus (**e**) levels.

An average number of sequence reads of 37,077 and an average OTU number of 290 was obtained by high-throughput sequencing for planktonic fungi in the 15 experimental groups (Table 2). The analysis showed that the planktonic fungal communities comprised five known fungal phyla: Ascomycota, Rozellomycota, Basidiomycota, Chytridiomycota, and Zygomycota (Fig. 4a).

In addition, a large number of sequences were Unclassified Fungi and NorankFungi, with the former accounting for 10.13–98.16% of the total OTUs in the samples tested. Among the five known fungal phyla observed, Ascomycota and Basidiomycota accounted for the highest proportion in samples from Qushou, Kuxin, Songgang and Heijizui, with Ascomycota accounting for 46.49%, 22.05%, 61.32% and 24.67%, respectively, and Basidiomycota for 3.25–11.75% in the total communities. In the sample from Taizishan, Ascomycota, Chytridiomycota and Basidiomycota accounted for 0.85%, 0.69% and 0.34% of the observed OTUs.

Planktonic fungi in other taxa were also analyzed. The samples from Qushou included OTUs from 5 phyla, 21 classes, 48 orders, 91 families, and 107 genera, and those from Kuxin included 4 phyla, 18 classes, 38 orders, 73 families, and 83 genera. Songgang samples contained 4 phyla, 19 classes, 39 orders, 86 families, and 102 genera, Heijizui samples contained 5 phyla, 21 classes, 44 orders, 94 families, and 124 genera, and Taizishan samples contained 4 phyla, 14 classes, 16 orders, 42 families and 43 genera (Fig. 4).

### Correlation analysis of the planktonic fungal community and environmental factors

**Figure 5:**
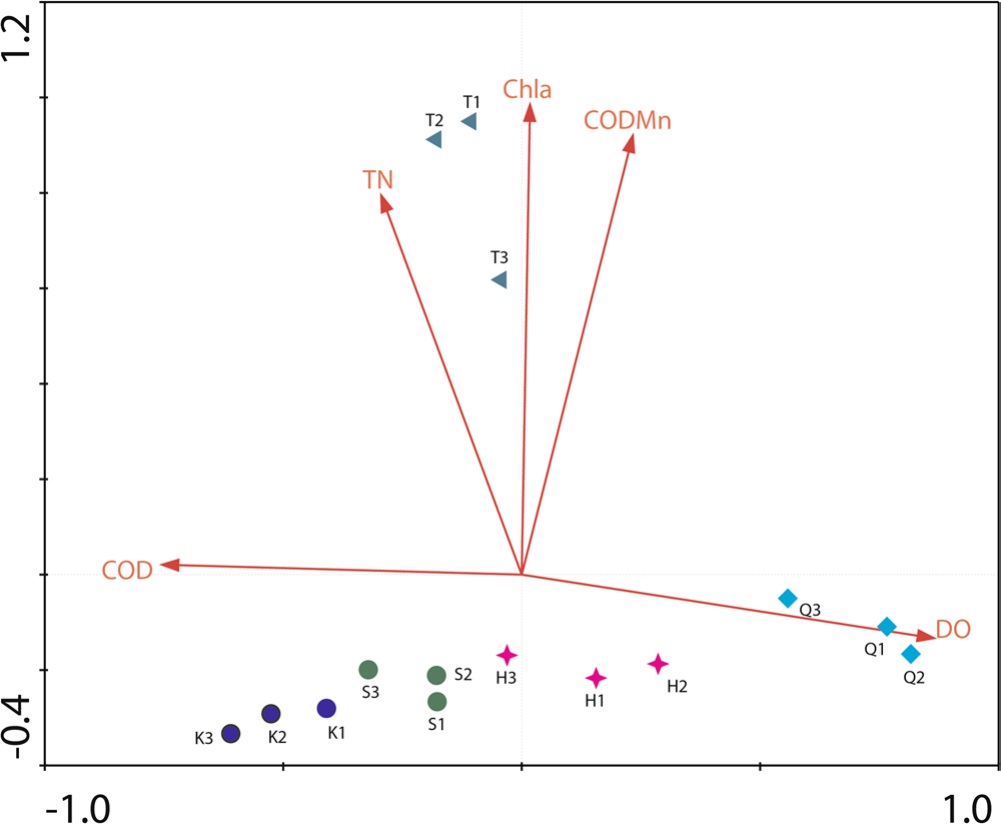
RDA of planktonic fungal communities and physicochemical water quality parameters.

Detrended correspondence analysis (DCA) showed that the longest gradient length for the four axes was less than 3.0 (2.847); thus, RDA in the linear model was selected for subsequent analyses. Insignificant variables were removed by a forward selection procedure, together with Monte Carlo permutation tests (n = 499 restricted permutations for time series). The RDA results are shown in Fig. 5. The physico-chemical properties with high correlation on the first-order axis were DO (R = 0.8405) and COD (R = −0.7331) and on the second-order axis were TN (R = 0.7809), Chl a (R = 0.9678), and COD_Mn_ (R = 0.9041). The above analysis showed that TN, Chl a, COD_Mn_, DO and COD were significantly correlated with the planktonic fungal community structure (*P* < 0.05) and the important factors affecting the distribution of planktonic fungi.

To elucidate the factors influencing the diversity of the planktonic fungal communities, Chao1 and ACE community abundance indices, Shannon and Simpson community diversity indices, library coverage, and physico-chemical water quality parameters were used for Spearman correlation analysis. The results showed TN, COD, and DO to be significantly correlated with the diversity indices, with TN and COD being negatively correlated with OTU number, Shannon, ACE, and Chao1, whereas COD was positively correlated with the Simpson index. In contrast, DO and diversity indices were significantly positively correlated with OTU number, Shannon, ACE, and Chao1 and significantly negatively correlated with the Simpson index (Table 3). Spearman correlation analysis between TN, COD, and DO showed that TN correlated positively with COD (R = 0.523, *P* = 0.046) and negatively with DO (R = −0.583, *P* = 0.022) (Table S3).

**Table 3:** Spearman correlation analysis between community richness and diversity indices and physicochemical water quality parameters.

	OTUs	Shannon	Simpson	Ace	Chao1	Coverage
NO_3_-N	−0.525*	−0.625*	0.346	−0.328	−0.453	0.038
Chla	−0.136	−0.109	−0.282	−0.023	−0.105	0.138
T	−0.246	−0.096	−0.221	−0.239	−0.275	0.546*
pH	−0.245	−0.270	0.375	−0.424	−0.325	0.259
DO	0.517*	0.515*	−0.701**	0.597*	0.559*	−0.279
COD_Mn_	−0.282	−0.228	−0.137	−0.309	−0.305	0.313
COD	−0.818**	−0.882**	0.918**	−0.775**	−0.800**	0.014
TN	−0.732**	−0.680**	0.496	−0.774**	−0.774**	0.521*
NH_4_-N	−0.313	−0.307	0.229	−0.433	−0.377	0.449
TP	−0.338	−0.320	0.047	−0.334	−0.315	0.175

*Indicates significant differences (*P* < 0.05).

**Indicates extremely significant differences (*P* < 0.01).

To further clarify the correlations between different planktonic fungal taxa and environmental factors, Spearman correlation analysis was performed for planktonic fungal phyla and families and physical and chemical water quality parameters. Correlation analysis between planktonic fungal phyla and physico-chemical parameters showed that Ascomycota was negatively correlated with Chl a (R = −0.631, *P* = 0.012) and NO_3_-N (R = −0.738, *P* = 0.002). Moreover, Basidiomycota was negatively correlated with NO_3_-N (R = −0.681, *P* = 0.002), TN (R = −0.689, *P* = 0.004), and COD (R = −0.775, *P* = 0.001), and Chytridiomycota was negatively correlated with COD (R = −0.521, *P* = 0.046) and pH (R = −0.665, *P* = 0.007) and positively correlated with Chl a (R = 0.043, *P* = 0.012) and DO (R = 0.518, *P* = 0.048). Zygomycota was negatively correlated with COD (R = −0.583, *P* = 0.023), and Unclassified Fungi was positively correlated with Chl a (R = 0.813, *P* = 0.001) and NO_3_-N (R = 0.713, *P* = 0.003).

**Figure 6:**
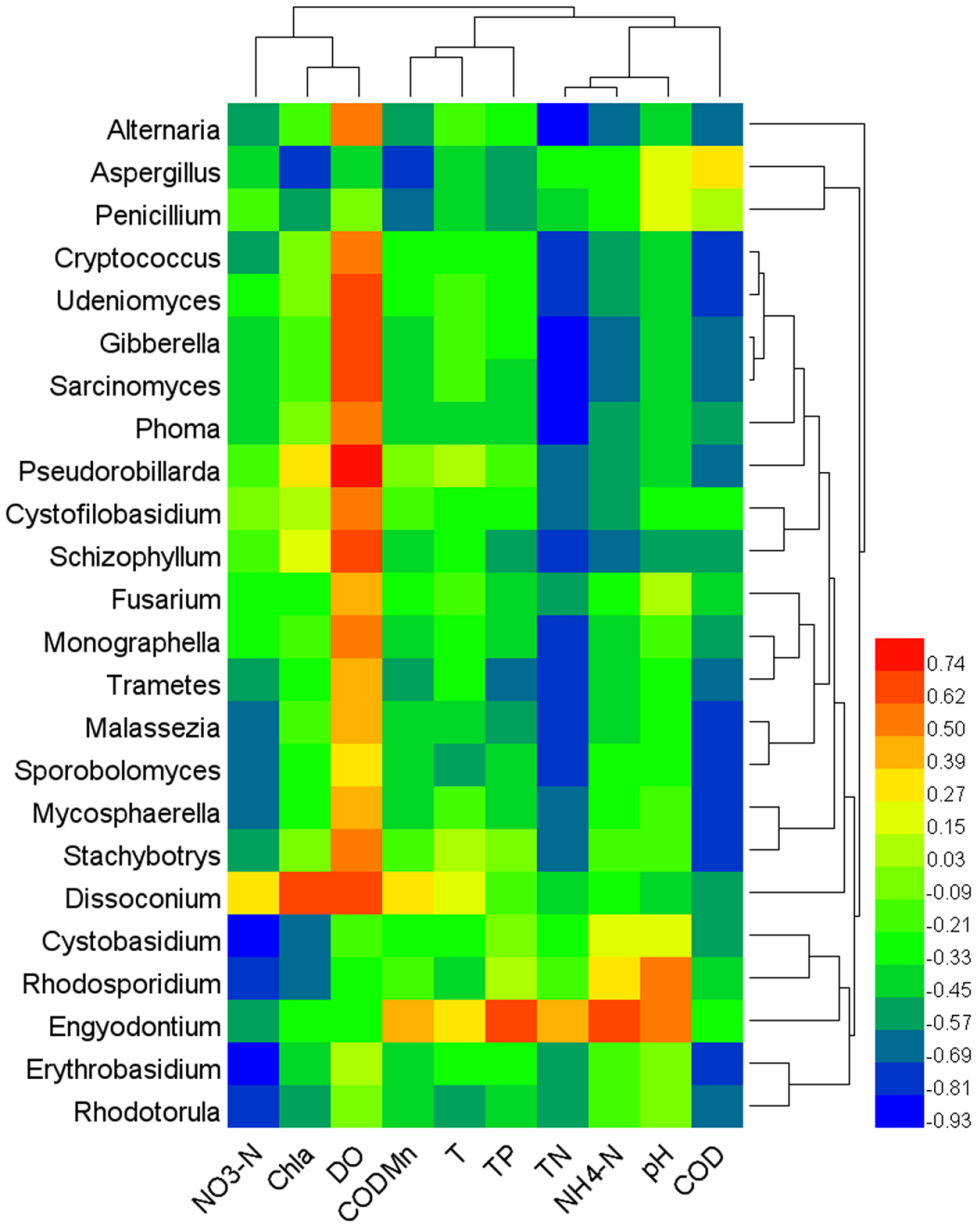
Heat map and hierarchical clustering tree of Spearman correlation analysis of planktonic fungal families and physicochemical water quality parameters.

According to correlation analysis between the observed planktonic fungal families and physico-chemical water quality parameters, TN, COD and DO were the major parameters related to the planktonic fungal community at the family level, with TN and COD exhibiting a negative correlation and DO a generally positive correlation (Fig. 6). The families Mycosphaerellaceae, Erysiphaceae, Davidiellaceae, Sclerotiniaceae and Dothioraceae in the phylum Ascomycota and the families Malasseziaceae, Norank Erythrobasidiales and Polyporaceae in the phylum Basidiomycota were significantly negatively correlated with TN and COD (*P* < 0.05). In addition, Pleosporaceae, Norank Pleosporales, Unclassified Ascomycota, Nectriaceae and Unclassified Dothideomycetes of some Ascomycota and Norank Tremellales and Cystofilobasidiaceae of some Basidiomycota were significantly negatively correlated with TN and COD while being significantly positively correlated with DO (*P* < 0.05). Norank Hypocreales, Dothioraceae, and Davidiellaceae in Ascomycota and Norank Sporidiobolales, Norank Erythrobasidiales and Cystobasidiaceae in Basidiomycota were extremely significantly negatively correlated with NO_3_-N (*P* < 0.01). Furthermore, Pleosporaceae, Norank Pleosporales and Unclassified Dothideomycetes in Ascomycota and Schizophyllaceae and Unclassified Malasseziales in Basidiomycota were significantly negatively correlated with NH_4_-N (*P* < 0.05), and Trichocomaceae and Norank Hypocreales in Ascomycota and Norank Sporidiobolales and Cystobasidiaceae in Basidiomycota were significantly negatively correlated with Chl a (*P* < 0.05). Regarding COD_Mn_, Trichocomaceae, Davidiellaceae and Unclassified Dothideomycetes in Ascomycota and Polyporaceae in Basidiomycota showed significantly negative correlations (*P* < 0.05).

## Discussion

### Composition of planktonic fungal community in the Danjiangkou Reservoir

Although information on fungal communities in reservoir ecosystems is rare, planktonic fungi are important components of aquatic ecosystems^14–16^. Li *et al*. used traditional culture-dependent methods to investigate the composition of planktonic fungal communities in coastal habitats off Pearl River Delta of China and found a preponderance of Ascomycota and Basidiomycota^35^. However, because most microorganisms cannot be purely cultured, modern culture-free molecular biology techniques have been applied to study planktonic fungi. To understand the composition of the planktonic fungal community in the surface water of the Danjiangkou Reservoir area, five monitoring sites were explored using the Illumina MiSeq sequencing platform. The planktonic fungal communities of Danjiangkou Reservoir primarily consisted of 7 phyla, including Ascomycota, Rozellomycota, Basidiomycota, Chytridiomycota, and Zygomycota, as well as Unclassified and Norank Fungi, comprising 294 genera, including *Aspergillus*, *Unclassified Chytridiomycota*, *Alternaria*, *Unclassified Davidiella*, *Malassezia*, *Mycosphaerella*, *Unclassified Pleosporales*, *Cryptococcus*, *Talaromyces*, *Penicillium*, and *Schizophyllum*. These results highlight the rich diversity of the composition of this community. Among known fungal phyla, Ascomycota and Basidiomycota were present in relatively high proportions, which is consistent with previous findings on the composition of planktonic fungi in lakes, oceans and other environments^16,21,35,36^. The results of our high-throughput sequencing analysis showed that the planktonic fungi in the surface water of the Danjiangkou Reservoir consisted of 7 phyla and 294 genera. Compared with the 3 phyla and 17 genera detected by Li *et al*. using traditional culture-depending methods^35^, as well as the 3 phyla and 15 genera that Kagami *et al*. observed using PCR-DGGE^20^, the high-throughput sequencing method used in this study provided much information on the planktonic fungal community, emphasizing the advantages of this technique. The results of this study comprehensively and accurately demonstrate the composition of the planktonic fungal community in the Danjiangkou Reservoir area and provide an important foundation for the management and maintenance of water quality in this area.

### Relationship between planktonic fungi and physicochemical water quality parameters

Because of their sensitivity to changes in water quality, aquatic microbes are important to biological monitoring and are used to monitor and evaluate water quality status^5–7^. In recent years, high-throughput sequencing technology has been widely used to biomonitor water quality because it can obtain specific DNA fragments and comprehensively display the compositional structure of biomes^22,23^. However, studies have rarely reported on the composition of planktonic fungal communities or analyzed the relationship of these communities with water quality in reservoir ecosystems using high-throughput sequencing technology^14,17,19^. Since the start of the Middle Section of the South-to-North Water Diversion Project, the normal water level of the Danjiangkou reservoir has risen from 157 m to 170 m, and the water ecosystem is in the process of reconstruction. Therefore, it is necessary to examine the factors influencing the planktonic fungal communities in the Danjiangkou Reservoir. Here, the composition of the planktonic fungal community at five monitoring sites in the Danjiangkou Reservoir area was investigated using high-throughput sequencing. RDA of the planktonic fungal community and environmental factors in the Danjiangkou Reservoir area revealed TN, Chl a, COD_Mn_, DO and COD to be important factors influencing the distribution of planktonic fungi. Subsequent Spearman correlation analysis between the composition of the planktonic fungal community and its diversity indices and physical and chemical water quality parameters showed that the impacts of TN, COD, and DO were the most significant. The Danjiangkou Reservoir area has the long-term problem of elevated TN due to pollution from agricultural non-point sources, industrial wastewater, and domestic sewage from villages and towns^27,33,34^. In aquatic ecosystems, microorganisms are the most important contributors to nitrogen circulation. Taylor and Cunliffe used high-throughput sequencing technology to study the composition of the coastal planktonic fungal community and its influencing factors in the United Kingdom, observing that it was primarily affected by salinity, nitrogen, and temperature^16^. In the present study, TN contents at Taizishan and Songgang exceeded 1.00 mg/L, which is the standard limit for Grade IV surface water; subsequent correlation analysis of the planktonic fungal communities and environmental factors showed that TN was an important factor influencing the distribution of planktonic fungi. The phylum Ascomycota was significantly negatively correlated with NO_3_-N, whereas Basidiomycota was significantly negatively correlated with TN and NO_3_-N. COD is an important comprehensive indicator used to evaluate the relative contents of organic pollutants in water bodies and has a significant impact on the microbial community composition of water bodies. Zhang *et al*. reported that the composition of planktonic bacterial communities in ponds was primarily affected by COD and TP^37^. For the Danjiangkou Reservoir, the correlation coefficient between COD and TN was 0.523, a significant positive correlation (*P* < 0.05), with COD having an effect similar to that of TN on the planktonic fungal community (Table 3). The concentration of DO can reflect the degree of pollution of a water body, especially organic matter pollution, and is an important index used to measure water quality because it is an important factor affecting the microorganisms in a water body^38^. Spietz *et al*. revealed a significant negative correlation between the diversity of planktonic bacteria and DO in the Hood Canal in the United States and reported that the composition of planktonic bacteria was primarily affected by DO^39^. In our study, correlation analysis of DO and other physical and chemical water quality indicators indicated that DO was negatively correlated with COD, TN, NH_4_-N, and TP in the Danjiangkou Reservoir, with COD, TN, and NH_4_-N reaching a level of significance (*P* < 0.05). These findings show that the impact of DO on the planktonic fungal community was opposite to that of TN and COD (Table 3). Pleosporaceae, Norank Pleosporales, Unclassified Ascomycota, Nectriaceae and Unclassified Dothideomycetes in the phylum Ascomycota and Norank Tremellales and Cystofilobasidiaceae in the phylum Basidiomycota were significantly negatively correlated with TN and COD and significantly positively correlated with DO (*P* < 0.05).

## Conclusion

Information on fungal communities in reservoir ecosystems is currently lacking. In this study, the community composition, related relationships and influencing factors of the planktonic fungi community in the Danjiangkou Reservoir were investigated. MiSeq sequencing was applied to comprehensively and accurately assess the composition of the planktonic fungi community in the Danjiangkou reservoir area. It was found that the planktonic fungi community in this area mainly consists of 7 phyla, including Ascomycota, Rozellomycota and Basidiomycota, and 294 genera, showing rich diversity of community composition. Analysis of diversity indices of the planktonic fungi community and physical and chemical parameters of water quality showed that the planktonic fungal community in the Danjiangkou Reservoir is sensitive to water quality parameters, providing the basis for utilizing planktonic fungi as indicators of changes in the water quality of reservoirs.

## Electronic supplementary material

Supplementary Materials
