# Supplementary material for: Planktonic fungal community structures and their relationship to water quality in the Danjiangkou Reservoir, China
Source: Sci Rep. 2018 Jul 13;8:10596. doi: 10.1038/s41598-018-28903-y (PMC6045663; doi:10.1038/s41598-018-28903-y)
Supplement: Supplementary file 1 — Supplementary Materials [file 41598_2018_28903_MOESM1_ESM.docx]

**Supporting Materials**

**Planktonic fungal community structures and their relationship to water quality in the Danjiangkou Reservoir, China**

Zhaojin Chen^1^, Jian Yuan^1^, Feng Sun^1^, Fei Zhang^1^, Yan Chen^2^, Chuanyu Ding^1^, Jianwei Shi^3^, Yuying Li^1^ & Lunguang Yao^1^

^1^ Collaborative Innovation Center of Water Security for Water Source Region of Mid-line of South-to-North Diversion Project of Henan Province, School of Agricultural Engineering, Nanyang Normal University, Nanyang 473061, P.R. China

^2^ School of Life Science and Technology, Nanyang Normal University, Nanyang 473061, P.R. China

^3^ Emergency Centre for Environmental Monitoring of the Canal Head of Middle Route Project of South-North Water Division, Xichuan 474475, P.R. China

Correspondence and requests for materials should be addressed to Z.C. (email: zhaojin_chen@163.com), Y.L. (email: lyying200508@163.com) or L.Y. (email: lunguangyao@163.com)

**The trophic level index (TLI) was calculated according to the following equations:**

*TLI(*$\sum)$*=* $\sum_{j=1}^{m} W_{j}\cdot$ *TLI(j)*  (1)

where TLI(∑) is integrated trophic level index; *W_j_* is correlative weighted score for trophic level index of *j*.

*W_j_= r_ij_^2^/*$\sum_{j=1}^{m} r_{ij}^{2}$ (2)

where *r_ij_* is relative coefficient, TLI (*j*) is trophic level index of *j*, *j* represents chlorophyll *a* (Chl *a*), TP, TN, COD_Mn_, and SD, respectively, which is expressed as the following formula.

*TLI (Chl a)=10(2.5+1.086lnChla)* (3)

*TLI (TP)=10(9.436+1.624lnTP)*  (4)

*TLI (TN)=10(5.453+1.694lnTN)* (5)

*TLI (COD_Mn_)=10(0.109+2.66lnCOD_Mn_)* (6)

*TLI (SD)=10(5.118-1.94lnSD)* (7)

Table S1. Correlations between the trophic state index and the level of the lake water quality.

Table S2. The classification standard of lake water quality and water function in China (GB3838-2002).

Table S3. Spearman correlation analysis between physico-chemical water quality parameters.

* indicates significant differences (*P*<0.05)

** indicates extremely significant differences (*P*<0.01)

Fig. S1. Hierarchical clustering tree created using UPGMA at the level of OTU.

Table S1.

| Trophic state | Trophic state index | Water quality |
| --- | --- | --- |
| Oligotrophic | 0<TLI≤30 | Excellent |
| Mesotrophic | 30<TLI≤50 | Good |
| Eutrophic | 50<TLI≤60 | Polluted |
| Supereutrophic | 60<TLI≤70 | Superpolluted |
| Hypereutrophic | 70<TLI≤100 | Hyperpolluted |

Table S2.

| Water classification | Water function | DO (mg/L) (≥) | TN (mg/L)  (≤) | NH_4_-N  (mg/L) (≤) | TP (mg/L) (≤) | COD (mg/L) (≤) | COD_Mn_  (mg/L) (≤) |
| --- | --- | --- | --- | --- | --- | --- | --- |
| Class I | Applicable to water source and state reserve | 7.5 | 0.2 | 0.15 | 0.01 | 15 | 2 |
| Class II | Applicable to centralized source of drinking water, hydrobiology habitat and etc. | 6 | 0.5 | 0.5 | 0.025 | 15 | 4 |
| Class III | Applicable to centralized source of drinking water, aquaculture and etc. | 5 | 1.0 | 1.0 | 0.05 | 20 | 6 |
| Class IV | Applicable to industry and recreation | 3 | 1.5 | 1.5 | 0.1 | 30 | 10 |
| Class V | Applicable to agriculture and landscape | 2 | 2.0 | 2.0 | 0.2 | 40 | 15 |

Table S3.

|  | NO_3_-N | Chla | T | pH | DO | COD_Mn_ | COD | TN | NH_3_-N | TP |
| --- | --- | --- | --- | --- | --- | --- | --- | --- | --- | --- |
| NO_3_-N | 1.000 | 0.669** | 0.276 | -0.180 | 0.125 | 0.384 | 0.511 | 0.370 | -0.099 | -0.012 |
| Chla | 0.669** | 1.000 | 0.350 | -0.439 | 0.558* | 0.624* | -0.123 | 0.093 | -0.097 | 0.160 |
| T | 0.276 | 0.350 | 1.000 | 0.266 | 0.306 | 0.473 | 0.029 | 0.415 | 0.134 | 0.309 |
| pH | -0.180 | -0.439 | 0.266 | 1.000 | -0.512 | 0.280 | 0.388 | 0.623* | 0.703** | 0.345 |
| DO | 0.125 | 0.558* | 0.306 | -0.512 | 1.000 | 0.077 | -0.527* | -0.583* | -0.599* | -0.051 |
| COD_Mn_ | 0.384 | 0.624* | 0.473 | 0.280 | 0.077 | 1.000 | -0.033 | 0.649** | 0.630* | 0.589* |
| COD | 0.511 | -0.123 | 0.029 | 0.388 | -0.527* | -0.033 | 1.000 | 0.523* | 0.113 | 0.085 |
| TN | 0.370 | 0.093 | 0.415 | 0.623* | -0.583* | 0.649** | 0.523* | 1.000 | 0.784** | 0.412 |
| NH_3_-N | -0.099 | -0.097 | 0.134 | 0.703** | -0.599* | 0.630* | 0.113 | 0.784** | 1.000 | 0.519* |
| TP | -0.012 | 0.160 | 0.309 | 0.345 | -0.051 | 0.589* | 0.085 | 0.412 | 0.519* | 1.000 |


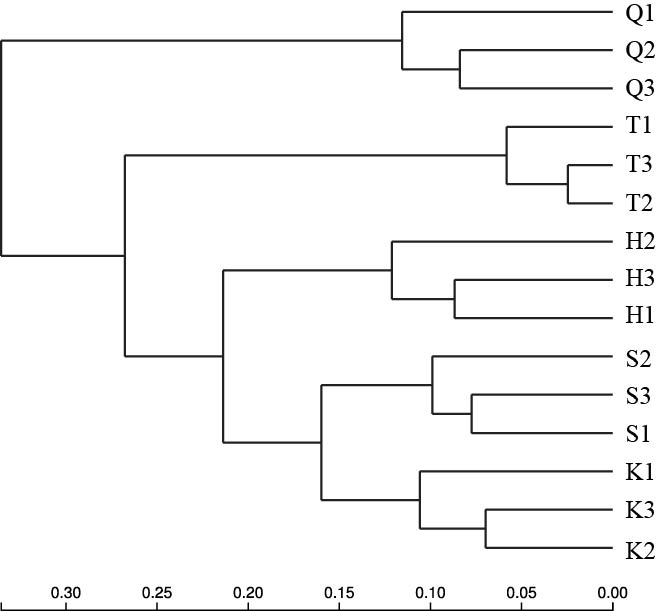


Fig. S1.
